# Monocyte Subsets, Stanford-A Acute Aortic Dissection, and Carotid Artery Stenosis: New Evidences

**DOI:** 10.1155/2019/9782594

**Published:** 2019-08-06

**Authors:** Noemi Cifani, Maria Proietta, Maurizio Taurino, Luigi Tritapepe, Flavia Del Porto

**Affiliations:** ^1^Dipartimento di Medicina Clinica e Molecolare, Ospedale Sant'Andrea, Facoltà di Medicina e Psicologia, Ospedale Sant'Andrea, “Sapienza, ” Università di Roma, Italy; ^2^Dipartimento di Medicina Clinica e Molecolare, Ospedale Sant'Andrea, Facoltà di Medicina e Psicologia, Ospedale Sant'Andrea, UOS Aterosclerosi e Dislipidemia, “Sapienza, ” Università di Roma, Italy; ^3^Dipartimento di Medicina Clinica e Molecolare, Ospedale Sant'Andrea, Facoltà di Medicina e Psicologia, Ospedale Sant'Andrea, UOC Chirurgia Vascolare, “Sapienza, ” Università di Roma, Italy; ^4^Dipartimento di Scienze Anestesiologiche, Medicina Critica e Terapia del Dolore, Facoltà di Medicina e Odontoiatria, Policlinico Umberto I, “Sapienza, ” Università di Roma, Italy; ^5^Dipartimento di Medicina Clinica e Molecolare, Ospedale Sant'Andrea, Facoltà di Medicina e Psicologia, Ospedale Sant'Andrea, UOC Medicina Interna, “Sapienza, ” Università di Roma, Italy

## Abstract

Monocytes are a heterogeneous cell population distinguished into three subsets with distinctive phenotypic and functional properties: “classical” (CD14++CD16-), “intermediate” (CD14++CD16+), and “nonclassical” (CD14+CD16++). Monocyte subsets play a pivotal role in many inflammatory systemic diseases including atherosclerosis (ATS). Only a low number of studies evaluated monocyte behavior in patients affected by cardiovascular diseases, and data about their role in acute aortic dissection (AAD) are lacking. Thus, the aim of this study was to investigate CD14++CD16-, CD14++CD16+, and CD14+CD16++ cells in patients with Stanford-A AAD and in patients with carotid artery stenosis (CAS). *Methods*. 20 patients with carotid artery stenosis (CAS group), 17 patients with Stanford-A AAD (AAD group), and 17 subjects with traditional cardiovascular risk factors (RF group) were enrolled. Monocyte subset frequency was determined by flow cytometry. *Results*. Classical monocytes were significantly increased in the AAD group versus CAS and RF groups, whereas intermediate monocytes were significantly decreased in the AAD group versus CAS and RF groups. *Conclusions*. Results of this study identify in AAD patients a peculiar monocyte array that can partly explain depletion of T CD4+ lymphocyte subpopulations observed in patients affected by AAD.

## 1. Introduction

Atherosclerosis (ATS) is a multifactorial disease [1] characterized by an inflammatory remodeling of the arterial wall. Depending on size and site of vessels involved, ATS leads to a wide range of cardiovascular diseases (CVDs) [2], including ischemic heart disease, cerebrovascular disease, carotid artery stenosis (CAS), abdominal aortic aneurism (AAA), acute aortic dissection (AAD), and other conditions [3, 4]. Immune response strongly affects the outcome of intraparietal inflammation: T helper (Th) 1 lymphocytes have been mainly associated with plaque formation and Th2 lymphocytes with AAA, whereas macrophages have been related to AAD [1, 5, 6].

Monocytes represent the circulating precursor of tissue macrophages [7] and play an important role in atherogenesis, being rapidly attracted by activated endothelial cells [8]. During an atherosclerotic process, their differentiation into macrophages is associated with upregulation of phagocytic activity leading to lipid accumulation and formation of typical foam cells [1]. Monocytes are a heterogeneous cell population distinguished by the expression of the surface markers CD14 (coreceptor for LPS) and CD16 (receptor for Fc*γ*RIII) [9] into three subsets: “classical” (CD14++CD16-), “intermediate” (CD14++CD16+), and “nonclassical” (CD14+CD16++) [10]. Each monocyte subset possesses distinctive phenotypic and functional properties and displays different immune functions, distinguished by cytokine profiles and phagocytic activity [11]. A low number of studies evaluated monocyte behavior in patients affected by CVDs. Classical monocytes have been independently associated with cardiovascular events including death, myocardial infarction, and stroke [12, 13]. Furthermore, experimental evidences support the role of intermediate monocytes in atheroocclusive diseases [14], such as coronary artery disease (CAD) [15, 16], cardioembolic stroke [17], CAS [18], unstable angina [12, 18], and AAA [19]. However, to our knowledge, data about the role of monocyte subsets in AAD are still lacking.

Therefore, we evaluated CD14++CD16-, CD14++CD16+, and CD14+CD16++ cells in patients with Stanford-A AAD and in patients with CAS.

## 2. Materials and Methods

This was an observational retrospective study.

The population included in this study was composed of 17 patients undergoing Stanford-A AAD surgical repair at the Attilio Reale Heart and Great Vessels Department, Policlinico Umberto I, “Sapienza” University of Rome (AAD group). Patients were selected on the basis of the following inclusion criteria: (i) Stanford-A AAD; (ii) no history of neoplasm or autoimmune, infectious, or inflammatory systemic diseases; (iii) no presence of genetic syndromes known to be responsible for aortic disease; and (iv) no family history of aortic dissection or aneurysm.

A group of 20 patients with critical CAS (CAS group) was selected among those undergoing carotid thrombo-endo-arteriectomy (TEA) at the Department of Vascular Surgery, Sant'Andrea Hospital, “Sapienza” University of Rome. Patients were enrolled on the basis of the following inclusion criteria: (i) critical carotid stenosis, defined as a narrowing of the carotid lumen ≥ 70% [20, 21]; (ii) no cardiac causes of stroke; (iii) no history of neoplasm or autoimmune or inflammatory systemic diseases; and (iv) no familiar or personal history of aneurysms/dissection. All patients underwent physical and neurological examinations, carotid artery ultrasound, and angiography by magnetic resonance imaging (MRI) or contrast tomography (CT).

Seventeen patients with traditional cardiovascular risk factors attending the Department of Atherosclerosis and Dyslipidemia, Sant'Andrea Hospital, “Sapienza” University of Rome, were used as the control group (RF group). Patients were selected on the basis of the following criteria: (i) no acute cerebrovascular symptoms or history of cardiovascular disease, (ii) no carotid stenosis > 20%, and (iii) no familiar or personal history of aneurysms/dissection.

No significant differences regarding age (mean age ± SD: 68.83 ± 4.11 years, 59.85 ± 11.01 years, and 62.59 ± 11.08 years for CAS, RF, and AAD, respectively), sex, diabetes, hypertension, dyslipidemia, and body mass index (BMI) were observed between CAS and RF groups.

AAD patients were matched with CAS and RF patients for age, sex, diabetes, and BMI but not for hypertension and dyslipidemia.

A venous blood sample was withdrawn from each patient (just before surgery) and from each control, in order to isolate peripheral blood mononuclear cells (PBMCs) by density gradient centrifugation (Lympholyte, Cedarlane, Hornby, CA). (Since Attilio Reale Heart and great Vessels Department is an hub reference center for AAD, all patients underwent to surgery within 6 hours from the onset of the symptoms and blood samples were collected within this time).

**Figure 1:**
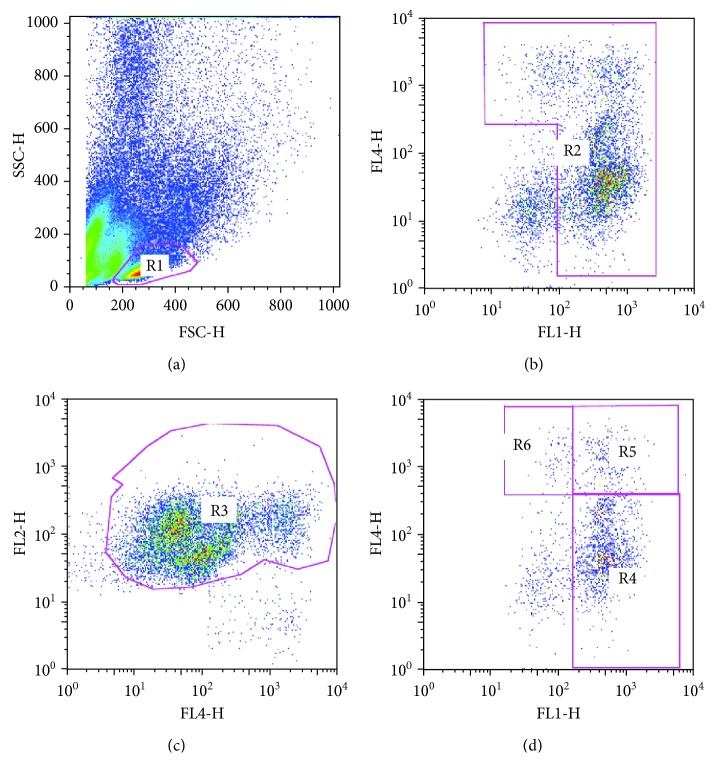
Representative flow cytometry strategy. Cells are visualized on FSC vs. SSC, and gate R1 is drawn around the monocyte cloud (a). These cells are then viewed on a CD14 (FITC, FL1-H) vs. CD16 (APC, FL4-H) plot, and gate R2 is drawn around the monocyte cloud (b). Gate R2 cells are viewed on a CD16 (APC, FL4-H) vs. HLA-DR (PE, FL2-H) plot, and HLA-DR-negative NK cells were excluded drawing R3 gate (c). Then, R3 monocyte population is viewed again on a CD14 (FITC, FL1-H) vs. CD16 (APC, FL4-H) plot, and CD14++CD16- (gate R4), CD14++CD16+ (gate R5), and CD14+CD16++ (gate R6) cells are defined according to the surface expression of CD14 and CD16 (d).

Monocyte subsets were analyzed by flow cytometry as previously described [22–24] using the following antibodies: CD14 FITC (BD Biosciences, San Jose, CA, USA), CD16 APC (BD Biosciences, San Jose, CA, USA), and HLA-DR PE (BD Biosciences, San Jose, CA, USA). Briefly, cells were first visualized on FSC vs. SSC, and an ample gate was drawn around the monocyte cloud to exclude the majority of debris and lymphocytes. These cells were then viewed on a CD14 vs. CD16 plot. Moreover, the presence of natural killer (NK) cells, most of which are CD16-positive and could interfere with CD16+ monocyte count, was checked by HLA-DR antibody; accordingly, HLA-DR-negative NK cells were excluded. Monocyte subsets CD14+CD16*−*, CD14+CD16+, and CD14+CD16++ were, therefore, defined according to the surface expression of CD14 and CD16 [23, 9] (Figure 1).

On the basis of the number of PBMC available, it was possible to test also CD4+ T lymphocytes in 6 patients of the CAS group, in 6 with RF, and in 10 with AAD. In the 10 AAD patients, immunohistochemistry of aortic specimens collected during surgery was performed as previously described [25].

FACS analysis was performed using a FACSCalibur cytometer (Becton-Dickinson) equipped with Cell Quest software. Isotype controls were used as compensation controls and to confirm antibody specificity.

All the statistical procedures were performed by GraphPad Prism 4 software (GraphPad Software Inc.).

The study was performed according to the principles of the Declaration of Helsinki and was approved by the ethics committee of the Faculty of Medicine.

Written informed consent was obtained from each patient or from an authorized family member.

## 3. Results and Discussion

### 3.1. Results

#### 3.1.1. Monocytes

**Figure 2:**
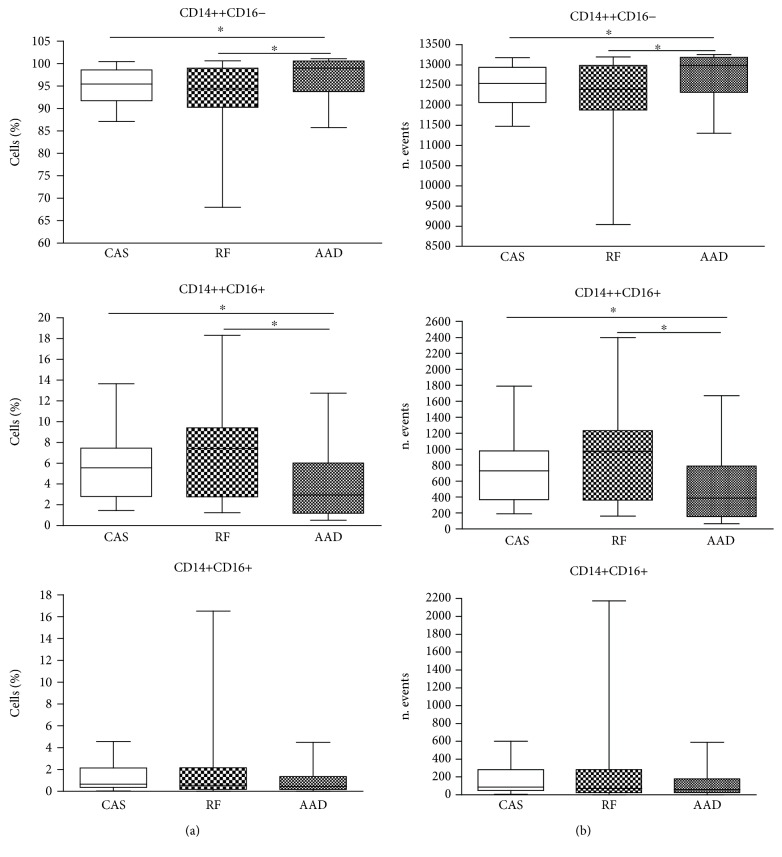
FACS analysis of monocyte subsets in CAS, RF, and AAD groups. Data were expressed as the percentage of cells (a) and the number of events (b). The 25 and 75 percentiles, median, minimal, and maximal are shown. Statistical analysis: Mann–Whitney nonparametric test. ^∗^*p* < 0.05.

Classical monocytes were significantly increased in the AAD group versus CAS and RF groups (*p* = 0.0342 and *p* = 0.0422, respectively), whereas intermediate monocytes were significantly decreased in the AAD group versus CAS and RF groups (*p* = 0.0494 and *p* = 0.0211, respectively). In particular, both intermediate and nonclassical monocytes progressively increased from AAD to RF, although any significant difference was observed regarding the nonclassical subset (Table 1, Figure 2). No significant differences were observed between CAS and RF groups for all monocyte subsets.

#### 3.1.2. Lymphocyte Subpopulations

A significant decrease of CD4+ T lymphocyte percentage (*p* = 0.05) was observed in AAD (mean ± SD: 31.04 ± 17.92; median: 30.00) versus CAS (mean ± SD: 50.63 ± 19.34; median: 55.35). No significant differences were observed between CAS and RF (mean ± SD38.43 ± 14.36; median 39.00) and between AAD and RF.

#### 3.1.3. Immunohistochemistry

Data regarding immunohistochemistry are reported in Table 2. In 8/10 AAD samples, an inflammatory infiltrate was observed within the aortic wall. In 7/8 samples, macrophages were the main population infiltrating the arterial wall, whereas only in one patient was observed a low infiltrate of T CD4+ lymphocytes.

### 3.2. Discussion

Our results demonstrated that patients affected by AAD show a peculiar monocyte pattern characterized by elevated classic and reduced intermediate cell subsets, which predispose them to a prevalent natural immune response. Moreover, we observed that CAS and AAD patients displayed an opposite monocyte array, confirming that immune response plays a pivotal role in driving atherosclerotic parietal remodeling toward occlusion or rupture. In this field, it has been demonstrated that a prevalent CD4+ immune response directs subintimal inflammation toward plaque formation, whereas a prevalent innate macrophage activation underlies medial degeneration and aortic rupture in Stanford-A AAD patients with no genetic predisposition [1, 6].

We, indeed, considered AAD and asymptomatic critical CAS as the opposite sides of the same ATS diseases, in which immune response drives parietal remodeling toward rupture or stable occlusion.

Monocytes represent a systemic reservoir of myeloid precursors for renewal of tissue macrophages and dendritic cells, but they also exert effector/antigen-presenting cell and regulatory functions. Macrophages are the main cells involved in the innate immune response and play a crucial role in the inflammatory process underlying ATS [26]. These cells, indeed, express an array of inflammatory factors, as well as matrix metalloproteinases (MMPs), which are responsible for maintaining intraparietal inflammation and degrading extracellular matrix [27]. Their activation has been related to myocardial infarction, stroke, and CAS [28]. Moreover, several studies indicated, both in mice and in humans, that macrophage recall and their activation represent key events in the early phases of AAD [6].

Interestingly, monocytes are able to trigger and polarize T cell-mediated immune response [29–31]. In particular, intermediate monocytes exert proinflammatory actions [10, 32] and have been reported to favor T cell differentiation toward Th1 and Th17 [33].

Experimental evidences support the role of intermediate monocytes in atheroocclusive diseases [22–24], such as symptomatic and asymptomatic CAS, cardioembolic stroke, and unstable angina [12, 17, 18]. Our results confirmed a high percentage of intermediate monocytes in CAS patients, whereas such subset was decreased in the AAD group versus both CAS and RF. This suggests that such depletion is specifically related to aortic rupture and can at least in part explain the lack of T CD4+ subpopulations which characterizes Stanford-A AAD [6, 34]. We, indeed, confirm that CD4+ T lymphocytes are significantly reduced in peripheral blood of AAD patients in comparison with CAS [6]. Moreover, a prevalent macrophage infiltrate was found within the tunica media in aortic samples, whereas T CD4+ lymphocytes were poorly represented.

In the AAD group, a significant increase of classic monocytes was documented versus both CAS and RF. Monocyte CD14++CD16- are mainly involved in natural response against pathogens. Furthermore, this subset has been related to the inflammatory process occurring in ATS [35]. The increase of such pattern in Stanford-A AAD patients strongly confirms that inflammation underlies also ascending aortic wall rupture in patients with no genetic predisposition and supports the hypothesis of a microbial contribution to AAD [36].

## 4. Conclusion

This study seems of particular interest, since to our knowledge, it is the first report about monocyte subsets in AAD. We found that Stanford-A AAD patients with no genetic predisposition display a peculiar monocyte pattern, which strongly differs from that observed in the CAS group. We, therefore, can speculate that monocytes, particularly CD14++CD16+ cells, can represent the link between innate and adaptive immunity and can contribute to drive immune response toward a matrix degrading natural response.

## Figures and Tables

**Table 1:** Percentage of monocyte subsets in CAS, AAD, and RF groups.

	Group CAS (*n* = 20) Mean ± SD	*M*	Group AAD (*n* = 17) Mean ± SD	*M*	Group RF (*n* = 17) Mean ± SD	*M*
Classical monocytes	93.05 ± 4.21	93.74	95.37 ± 4.04	97.11	91.11 ± 7.68	92.67
Intermediate monocytes	5.78 ± 3.59	5.45	3.69 ± 3.23	2.89	6.99 ± 4.96	7.25
Nonclassical monocytes	1.17 ± 1.20	0.65	0.94 ± 1.19	0.45	1.90 ± 3.84	0.54

**Table 2:** Immune infiltrate within the aortic wall.

Sample	Macrophages (CD68+)	Lymphocytes (CD4+)
1	—	1
2	1	1
3	1	—
4	—	—
5	3	—
6	—	—
7	2	—
8	3	1
9	3	2
10	1	—

## Data Availability

The data used to support the findings of this study are available from the corresponding author upon request.
